# South American precipitation dipole forced by interhemispheric temperature gradient

**DOI:** 10.1038/s41598-022-14495-1

**Published:** 2022-06-22

**Authors:** Marília C. Campos, Cristiano M. Chiessi, Valdir F. Novello, Stefano Crivellari, José L. P. S. Campos, Ana Luiza S. Albuquerque, Igor M. Venancio, Thiago P. Santos, Dayane B. Melo, Francisco W. Cruz, André O. Sawakuchi, Vinícius R. Mendes

**Affiliations:** 1 Institute of Geosciences, University of São Paulo, São Paulo, Brazil; 2 School of Arts, Sciences and Humanities, University of São Paulo, São Paulo, Brazil; 3 Department of Geosciences, University of Tübingen, Tübingen, Germany; 4 Graduate Program in Geochemistry, Fluminense Federal University, Niterói, Brazil; 5 MARUM—Center for Marine Environmental Sciences, University of Bremen, Bremen, Germany; 6 Institute of Marine Science, Federal University of São Paulo, Santos, Brazil

**Keywords:** Palaeoceanography, Palaeoclimate

## Abstract

Tropical South American hydroclimate sustains the world’s highest biodiversity and hundreds of millions of people. Whitin this region, Amazonia and northeastern Brazil have attracted much attention due to their high biological and social vulnerabilities to climate change (i.e. considered climate change hotspots). Still, their future response to climate change remains uncertain. On precession timescale, it has been suggested that periods of decreased western Amazonian precipitation were accompanied by increased northeastern Brazilian precipitation and vice-versa, setting an east–west tropical South American precipitation dipole. However, the very existence of this precession-driven precipitation dipole remains unsettled given the scarcity of long and appropriate northeastern Brazilian records. Here we show that the precession-driven South American precipitation dipole has persisted over the last 113 ka as revealed by a northern northeastern Brazilian precipitation record obtained from quartz thermoluminescence sensitivity measured in marine sediment cores. Precession-induced austral summer insolation changes drove the precipitation dipole through the interhemispheric temperature gradient control over the regional Walker circulation and the Intertropical Convergence Zone seasonal migration range. Since modern global warming affects the interhemispheric temperature gradient, our study provides insights about possible future tropical South American hydroclimate responses.

## Introduction

**Figure 1:**
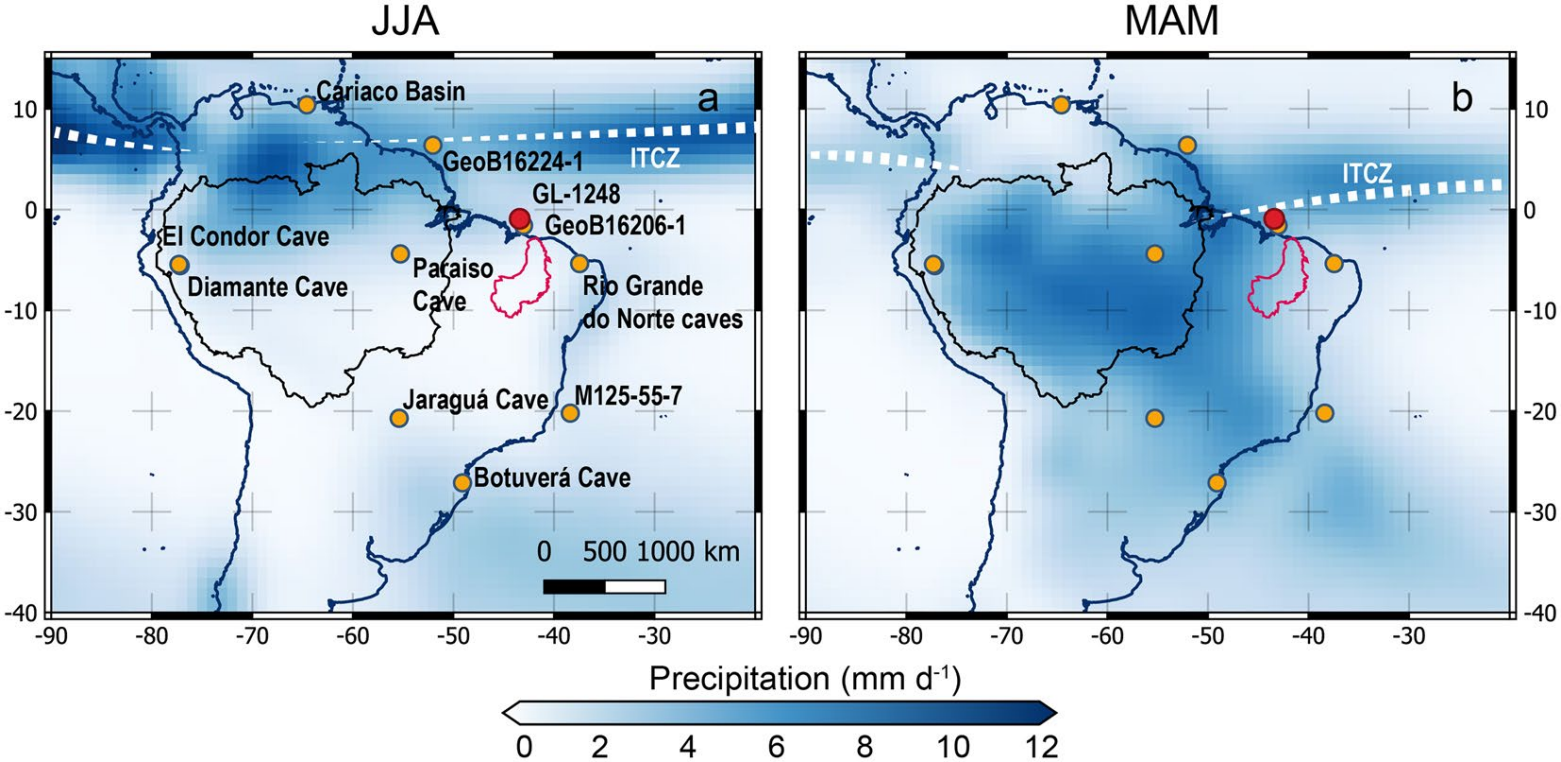
Geographic region of interest for this study and modern climatological precipitation. (**a**) Location of the hydroclimate records (red and yellow circles) discussed herein and June–August (JJA) average precipitation (color shading)^40^. (**b**) Same as (**a**) but for March–May (MAM) average precipitation. White dashed band in panels (**a**) and (**b**) represents the position of the Intertropical Convergence Zone (ITCZ) during JJA and MAM, respectively. Red and black area in panels (**a**) and (**b**) represent the location of the Parnaíba River drainage basin and Amazon Basin, respectively. The location of marine sediment core GL-1248 (this study) is marked with a red circle. The yellow circles mark the location of the flowing hydroclimate records: marine sediment cores MD03-2621 (Cariaco Basin)^28^, GeoB16224-1 (off Amazon Basin)^33^, GeoB16206-1 (off Parnaíba River drainage basin)^21^, M125-55-7 (off Doce River drainage basin)^31^; and speleothems records from Diamante^5^, Jaraguá^30^, Paraíso^14^, Rio Grande do Norte^6^ and Botuverá^29^ caves.

The response of tropical South American (SA) hydroclimate to future climate change remains uncertain^1,2^. This region includes two important climate change hotspots, namely Amazonia and northeastern Brazil^3^ (Fig. 1). While regional and global climate models have shown some agreement on decreasing precipitation over large portions of Amazonia, future trends for northeastern Brazil are less clear^1,2,4^. Periods of decreased precipitation over western (W) Amazonia have been identified on orbital timescale^5^ whereby a precession-driven east–west precipitation dipole has been suggested to occur between this region and northern northeastern Brazil (NEB)^5,6^. According to this suggestion, periods of low austral summer insolation (e.g. the early Holocene, at ca. 10 thousand years before present—ka BP) were related to decreased precipitation over W Amazonia while NEB experienced increased precipitation^5–8^. The opposite occurred during periods of high austral summer insolation (e.g. the late Holocene), i.e. precipitation increased over W Amazonia and decreased over NEB.

Western Amazonia hosts the core region of the South American Monsoon System (SAMS), which is the main atmospheric feature influencing precipitation over (sub)tropical SA to the east of the Andes^9–11^, with a mature phase occurring during late austral spring and summer^12^. The east–west tropical SA precipitation dipole has been usually attributed to the high sensitivity of the SAMS to precession-induced changes in austral summer insolation^5,6^. Periods of low (high) austral summer insolation resulted in weaker (stronger) SAMS convection over W Amazonia. This, in turn, resulted in weaker (stronger) subsidence over NEB, both regions being connected by a regional Walker circulation. The weaker (stronger) the SAMS, the weaker (stronger) the ascending and descending branches of the regional Walker circulation^13^.

The east–west tropical SA precipitation dipole was described based on the antiphase relationship observed for the last ca. 26 ka (i.e. last precession cycle) between speleothem stable oxygen isotopic (δ^18^O) records from Rio Grande do Norte caves (located in NEB; Figs. 1, 3f) and Diamante Cave (located in W Amazonia; Figs. 1, 3c)^5,6^. In these regions, speleothem δ^18^O values are assumed to mainly reflect precipitation amount^6^ and monsoon intensification^5^.

However, the existence of the east–west tropical SA precipitation dipole has been recently challenged based on a longer (ca. 45 ka) speleothem δ^18^O record from Paraíso Cave (located in eastern (E) Amazonia, Figs. 1, 3d)^14^. This δ^18^O record does not show precession-paced changes except for the Holocene. The authors invoked the effect of isotopic fractionation mediated by forest evapotranspiration to explain the differences between E and W Amazonian speleothem δ^18^O signals when comparing the last glacial to the Holocene, rather than precipitation amount driven by austral summer insolation (Fig. 3c,d). Major elemental ratios (e.g. Ti/Ca) from marine sediment cores collected off NEB^15–17^ neither show precession-paced precipitation changes, except for the last ca. 5.2 ka^18^. Since NEB hydroclimate records showing precession-paced precipitation changes only cover the last precession cycle (ca. 26 ka)^6,18^, the very existence of a precession-driven east–west tropical SA precipitation dipole remains unsettled. Additionally, changes in the seasonal range of the latitudinal migration of the Intertropical Convergence Zone (ITCZ) was recently suggested to also play a crucial role in controlling precession-scale precipitation over NEB^18^. Again, the short duration of the available records (ca. 5.2 ka)^18^ does not allow a long-term confirmation of precession-driven ITCZ changes. The ITCZ directly influences precipitation over NEB^19^ (Fig. 1). It shows a marked seasonal meridional migration according to the interhemispheric surface temperature gradient, i.e. when Northern Hemisphere (NH) surface temperature minus Southern Hemisphere (SH) surface temperature is positive (negative), the ITCZ migrates to the north (south), i.e. the ITCZ follows the warmer hemisphere^20^. In modern climate, precipitation over NEB peaks during late austral summer-early autumn, when the ITCZ reaches its southernmost position (Fig. 1b). Little precipitation occurs in the region during the rest of the year, when the ITCZ is at its northernmost position (i.e. over the Cariaco Basin, Fig. 1a) or between both extremes (i.e. between the southernmost and the northernmost positions).

**Figure 2:**
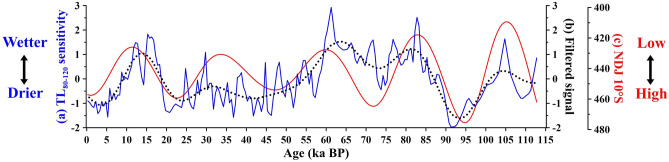
New northern NE Brazil (NEB) precipitation record. (**a**) Thermoluminescence (TL_80–120_) sensitivity of the 110 °C peak from marine sediment cores GL-1248 (0.92°S, 43.40°W) and GeoB16206-1 (1.58°S, 43.02°W)^21^ (details regarding merging these records are available in Text S1 and Fig. S1). (**b**) TL sensitivity record filtered in 0.026 ka frequency (19 ka, FIR filter) within software PAST v4.03^46^. (**c**) November–January (NDJ) insolation (W/m^2^) at 10°S^47^.

Here we present a precipitation record covering the last ca. 113 ka based on the quartz thermoluminescence (TL) sensitivity of marine sediment cores GL-1248 (0.92°S, 43.40°W, 2264 water depth, Fig. 1; Ref.^17^) and previously published GeoB16206-1 (1.58°S, 43.02°W, 1367 water depth, Fig. 1; Ref.^21^), collected off NEB (details regarding the preparation of the composite record are available in Text S1 and Fig. S1). During the last ca. 113 ka, these neighbor sites were influenced by the terrigenous discharge of the Parnaíba River^22,23^, the largest drainage basin in NEB (Fig. 1). Our composite record provides the hydroclimatic history of NEB for the last five precession cycles (Fig. 2a).

**Figure 3:**
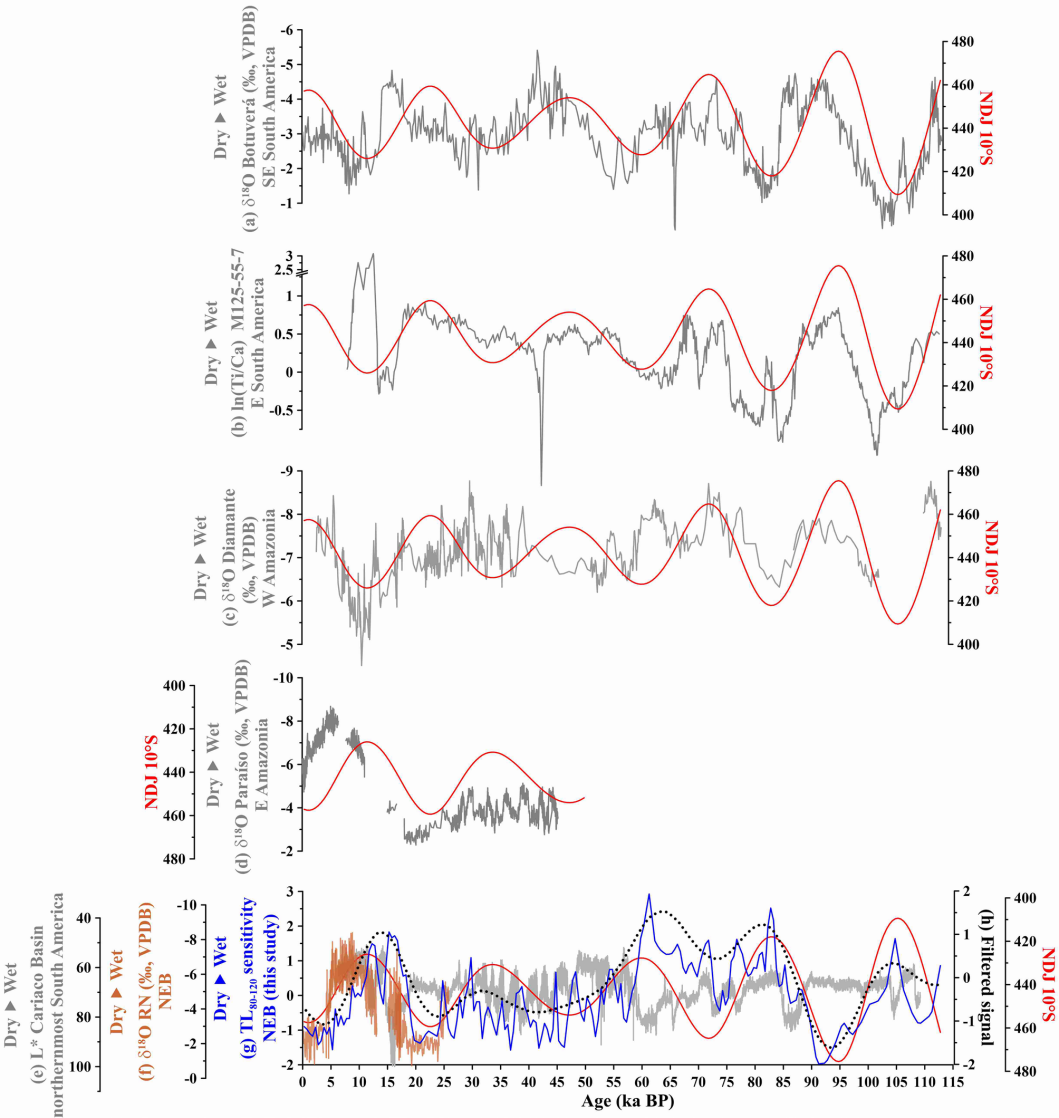
New northern NE Brazil (NEB) precipitation record together with other records from (sub)tropical South America. (**a**) Speleothem stable oxygen isotopic (δ^18^O) record from Botuverá Cave, southeastern (SE) South America (27.2°S, 49.2°W)^29^. (**b**) ln(Ti/Ca) from marine sediment core M125-55–7, off eastern (E) South America (20.4°S, 38.6°W; the axis was broken from 1.25 to 2.5)^31^. (**c**) Speleothem δ^18^O records from Diamante Cave, western (W) Amazonia (ca. 5°S, 77°W)^5^. (**d**) Speleothem δ^18^O record from Paraíso Cave, eastern (E) Amazonia (4.67°S, 55.45°W)^14^. (**e**) L* reflectance from the Cariaco Basin marine sediment core MD03-2621, off northernmost South America (10.7°N, 65°W)^28^. (**f**) Speleothem δ^18^O records from Rio Grande do Norte (RN) caves, NEB (5.6°S, 37.7°W)^6^. (**g**) Thermoluminescence (TL_80–120_) sensitivity of the 110 °C peak from marine sediment cores GL-1248 (0.92°S, 43.40°W) and GeoB16206-1 (1.58°S, 43.02°W)^21^, off NEB (this study). (**e**) TL record filtered in 0.026 ka frequency (19 ka, FIR filter) within software PAST v4.03^46^. Red curves are November–January (NDJ) insolation (W/m^2^) at 10°S^47^.

Our method is based on the TL sensitivity (i.e. light emitted per unit of mass and radiation dose) of fluvially transported quartz grains deposited in the western equatorial Atlantic. High (low) quartz TL sensitivity values indicate periods of increased (decreased) precipitation over the Parnaíba River drainage basin^21^ (further details regarding the proxy are provided in Text S2). The main advantages of this proxy are: (i) its fast response to changes in continental precipitation; (ii) the lack of post-depositional biases; and (iii) the absence of effects related to rainfall isotopic fractionation and changes in relative sea-level or biogenic carbonate production, which are important controllers of the previous mentioned paleoclimate archives on orbital timescale^24^.

Our 113 ka-long hydroclimate record^25^ shows a clear precession pacing (Figs. S3, S4), whereby periods of increased NEB precipitation (related to high TL sensitivity values) are coeval with periods of low austral summer insolation and vice-versa (Fig. 2). This is the first record from NEB covering more than the last precession cycle showing that the hydroclimate of this region is in antiphase with austral summer insolation. We suggest that precession-induced austral summer insolation changes drove NEB precipitation through the interhemispheric temperature gradient control over the Walker circulation and the ITCZ^18,20,26^.

**Figure 4:**
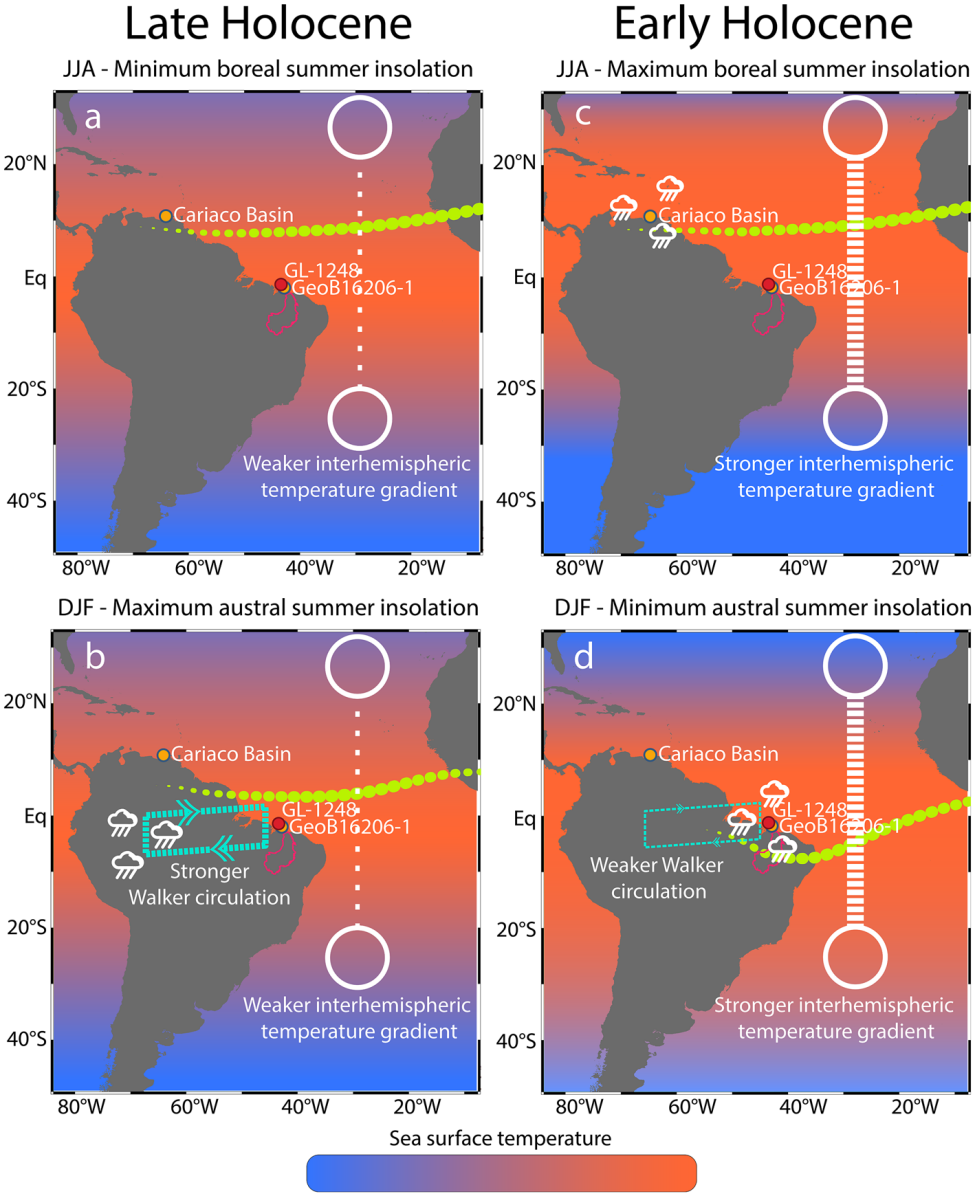
Schematic representation of the main climatological features and mechanism discussed herein. Herein, interhemispheric surface temperature gradient is represented by the difference between Northern Hemisphere (NH) surface temperature and Southern Hemisphere (SH) surface temperature (i.e. NH minus SH surface temperature). This temperature gradient is a result of the combination between insolation forcing and thermal inertia of both hemispheres. Since the SH has higher thermal inertia than NH, the SH thermal response to changes in insolation is slower than the NH. (**a**, **b**) represent periods of minimum boreal summer insolation (June, July and August—JJA) and maximum austral summer insolation (December, January and February—DJF), respectively, for the late Holocene. Late Holocene boreal and austral summers are marked by weak interhemispheric surface temperature gradient (represented by the white open circle connected through a dashed white arrow). It occurred because minimum insolation during boreal summer (**a**) makes the NH slight warmer than SH in JJA and maximum insolation during boreal winter (**b**) makes the NH slight colder than the SH during DJF. Because of the weak gradient, the ITCZ (neon green dotted band) migration to the north in JJA (**a**) and to the south in DJF (**b**) were not pronounced (i.e. contraction of the meridional range of the ITCZ). Additionally, periods of maximum austral summer insolation (**b**) were marked by stronger regional Walker circulation ascending and descending branches (dashed light green cell). Thus, contraction of the meridional range of the ITCZ and stronger regional Walker circulation resulted in drier conditions over northern NE Brazil (NEB) and northernmost South America (Cariaco Basin) and wetter conditions over the W Amazonia. (**c**, **d**) represent periods of maximum boreal summer insolation (JJA) and minimum austral summer insolation (DJF), respectively, for the early Holocene. Early Holocene boreal and austral summers are marked by strong interhemispheric surface temperature gradient (represented by the white open circle connected through a dashed white arrow). It occurred because maximum insolation during boreal summer (**c**) makes the NH strongly warmer than SH in JJA and minimum insolation during boreal winter (**d**) makes the NH strongly colder than the SH during DJF. Because of the strong gradient, the ITCZ (neon green dotted band) migration to the north in JJA (**c**) and to the south in DJF (**d**) was very pronounced (i.e. expansion of the meridional range of the ITCZ). Additionally, periods of minimum austral summer insolation (**d**) were marked by weaker regional Walker circulation ascending and descending branches (dashed light green cell). Thus, expansion of the meridional range of the ITCZ and weaker regional Walker circulation resulted in wetter conditions over NEB and northernmost South America (Cariaco Basin) and drier conditions over the W Amazonia.

For instance, during the early Holocene, the precession-induced orbital configuration caused a minimum in austral summer insolation and a maximum in boreal summer insolation. This pattern of summer insolation produced smaller changes in SH surface temperatures than in NH due to the higher SH thermal inertia. The different thermal inertia of both hemispheres translates into a more intense interhemispheric surface temperature gradient during austral and boreal summers of the early Holocene compared, for example, to the late Holocene, which is a period of precession-induced maximum austral summer insolation and minimum boreal summer insolation^18,27^ (Fig. 4).

In the early Holocene scenario, a weaker austral summer convection over the SAMS core region (which constitutes the ascending branch of the regional Walker circulation) decreased precipitation over W Amazonia (Fig. 4d). This occurred because minimum austral summer insolation reduced the ability of the continent to pull moisture from the ocean through the decreasing in the land–ocean thermal gradient, that overall reflects the difference in surface temperature between low latitudes and the NH mid-latitudes^26^. Similarly, the descending branch of the regional Walker circulation also became weaker as result of the decreased strength of the ascending branch, allowing precipitation to increase over NEB (Fig. 4d)^6^. Moreover, the stronger interhemispheric surface temperature gradient during austral and boreal summers forced a shift to the south of the southernmost limit of the ITCZ (Fig. 4d), and to the north of the northernmost limit of the ITCZ (Fig. 4c). Thus, during the early Holocene, an expansion of the seasonal range of the meridional ITCZ migration increased precipitation not only over NEB, located at the southernmost seasonal reach of the ITCZ, but also over northernmost SA (e.g. Cariaco Basin), located at the northernmost seasonal reach of the ITCZ (Fig. 4c,d). Indeed, records from the Cariaco Basin^28^ and NEB show in-phase precession-paced precipitation variability for the last ca. 113 ka (Fig. 3e–g). These ITCZ changes have been proposed to explain mid- to late Holocene variability in precipitation over NEB^18^. Our record demonstrates for the first time that this mechanism indeed operated on multiple precession cycles (Fig. 3g).

In the late Holocene scenario (Fig. 4a,b), the precession-induced maximum austral summer insolation resulted in stronger convection over the SAMS core region, increasing precipitation over W Amazonia (Fig. 4b). In turn, the subsidence over NEB strengthened, hampering precipitation in that region (Fig. 4b). Additionally, due to the more relaxed interhemispheric surface temperature gradient, the seasonal range of the meridional ITCZ migration contracted, further decreasing precipitation over NEB and northernmost SA (Figs. 3e–g, 4a,b)^5,6,18^.

In summary, a weaker (stronger) regional Walker circulation descending branch together with an expansion (contraction) of the meridional range of the ITCZ resulted in wetter (drier) conditions over NEB during periods of minimum (maximum) austral summer insolation (Figs. 3f,g; 4).

Our findings support the notion that speleothem δ^18^O values from different sides of Amazonia are recording different precipitation patterns within the basin, in which E Amazonia did not respond only to SAMS activity (Fig. 3c,d)^5^. This is also supported by the similar variability between W Amazonian δ^18^O records and the δ^18^O records from middle-western SA (e.g. Jaraguá Cave, Fig. 1) and southeastern SA (e.g. Botuverá Cave, Figs. 1 and 3a)^29,30^ as well as the Ti/Ca data from a marine sediment core collect off eastern SA (i.e. M125-55-7, Figs. 1 and 3b)^31^. These are regions where summer precipitation is related to the precession-driven SAMS intensity through source^29^ and/or amount^31,32^ effects. However, it is noteworthy that all hydroclimate reconstructions lose the strong insolation control between ca. 55 and 20 ka BP, except for southeastern SA speleothem records (Fig. 3b,c,g; further details regarding this topic are provided in Fig. S5 and Text S3).

This rationale regarding δ^18^O values from different sides of Amazonia is in disagreement with Wang et al. (Ref.^14^) that suggested a forest cover-driven isotopic fractionation effect over E and W Amazonian speleothem δ^18^O records. In this scenario, a less (more) densely vegetated Amazon Basin during a relatively drier (wetter) last glacial (early Holocene) climate would have reduced (increased) forest evapotranspiration, increasing (decreasing) the isotopic fractionation of incoming trade winds moisture from E to W Amazonia. Additional evidence supporting our rationale comes from a plant-wax δ^13^C record from a marine sediment core collected off the mouth of the Amazon River (i.e. GeoB16224-1; Fig. 1)^33^, which records an integrated signal from lowlands Amazonia. It shows that the Last Glacial Maximum is the period with the largest C3 vegetation cover within the interval 50–13 ka BP. Thus, the highest proportion of C3 vegetation and its related stronger evapotranspiration compared to C4 vegetation during the Last Glacial Maximum argue against the proposition of Wang et al. (Ref.^14^). The unclear orbital precipitation pattern in the Paraíso Cave δ^18^O record (i.e. E Amazonia) is likely related to the fact that precipitation over this region is in phase with both the SAMS and the ITCZ. The long duration of the rainy season at this region (7 months, from December to June^34^) may integrate different seasonal precipitation signals on orbital timescales resulting in a mixed response to austral summer insolation^26^.

**Figure 5:**
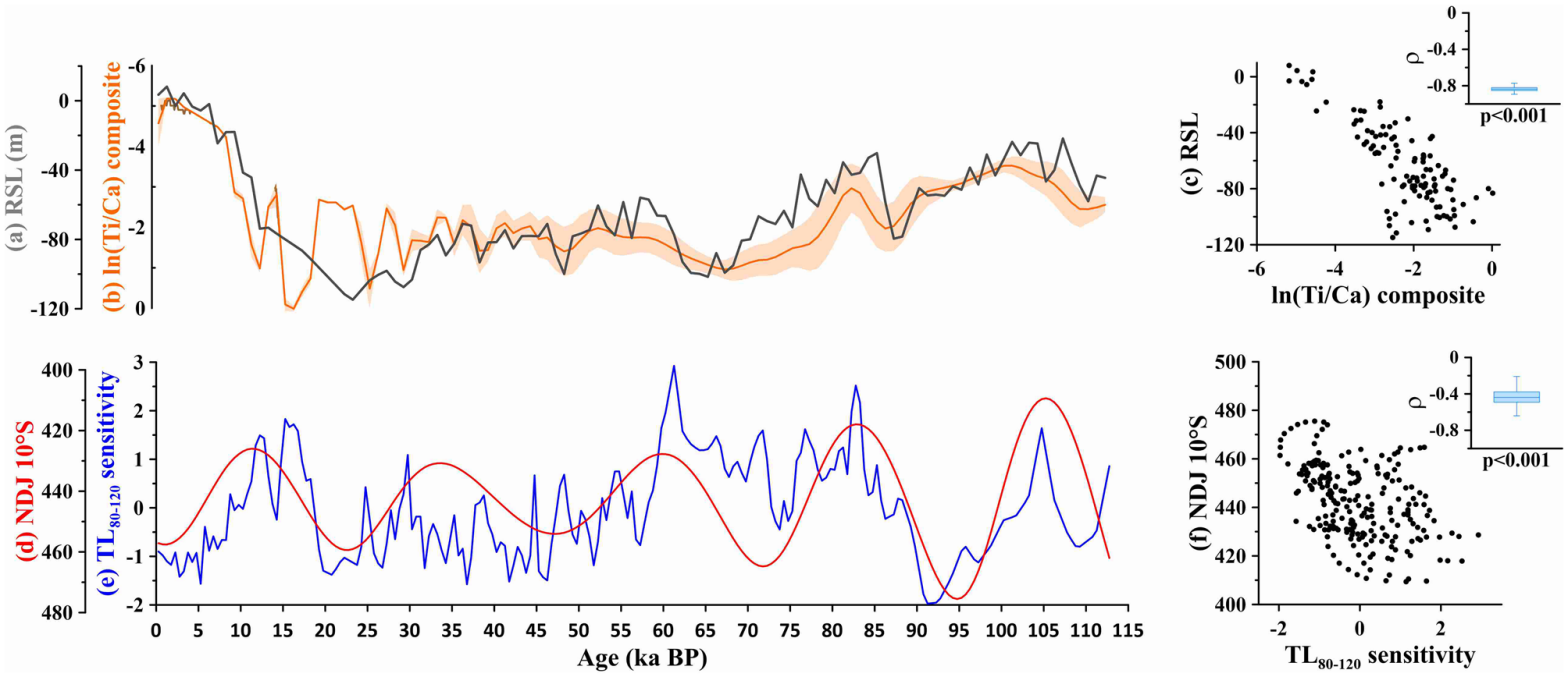
Northern NE Brazil (NEB) records and correlations with relative sea-level (RSL) and austral summer insolation. (**a**) RSL probability maximum (dark grey)^35^. (**b**) ln(Ti/Ca) composite record from marine sediment cores GL-1248^17^ and GeoB16206-1^48^ (orange curve) enveloped by standard deviation (orange shade). (**c**) Correlation between RSL record and ln(Ti/Ca) composite record (here we used the 500 year resolution chronological timeseries of the GL-1248 and GeoB16206-1 composite record described in Text S1). (**d**) November–January (NDJ) insolation (W/m^2^) at 10°S^47^. (**e**) Thermoluminescence (TL_80–120_) sensitivity of the 110 °C peak from marine sediment cores GL-1248 and GeoB16206-1^21^ (this study). (**f**) Correlation between NDJ insolation at 10°S^47^ and TL_80–120_ sensitivity record from marine sediment cores GL-1248 and GeoB16206-1.

Regarding NEB marine hydroclimate records longer than 5.2 ka, the reason for the absence of precession-paced precipitation is probably related to the narrow continental shelf which would make the region particularly sensitive to long-term changes in sea-level. Indeed, glacial-interglacial changes in the ln(Ti/Ca) from the composite record of marine sediment cores GeoB16206-1^7^/GL-1248^17^ respond remarkably well to relative changes in sea-level^35^ (Fig. 5a–c) (further details regarding other GL-1248 records are provided in Text S4 and Fig. S6). Since TL sensitivity only depends on continental processes (Text S2), this proxy only captures precession-paced changes in precipitation (Fig. 5d–f).

Finally, our precession-paced precipitation record shows that the hydroclimate of NEB was in antiphase with austral summer insolation and, thus, with W Amazonian precipitation, for the last 113 ka. Two factors were responsible thereof: (i) changes in the strength of the regional Walker circulation; and (ii) changes in the ITCZ seasonal migration range. These changes were notably forced by changes in the interhemispheric surface temperature gradient. Importantly, modern global warming is affecting the interhemispheric surface temperature gradient by warming more substantially the NH than the SH^2,36–39^. This shows some resemblance to the early Holocene situation described here. Thus, our study provides useful insights about possible tropical SA hydroclimate responses to such changes in interhemispheric surface temperature gradient.

## Methods

Global monthly precipitation shown in Fig. 1 was obtained from CPC Merged Analysis of Precipitation (CMAP) presented in Xie and Arkin (Ref.^40^), which has been constructed on a 2.5° latitude–longitude grid for the 17-year period from 1979 to 1995.

Marine sediment core GL-1248 was collected from the continental slope off NEB (0.92°S, 43.40°W, 2264 m water depth, 19.29 m long) by Petrobras (Fig. 1). Its age model is based on the combination of radiocarbon ages (based on planktonic foraminifera *Globigerinoides ruber* and *Trilobatus sacculifer*, handpicked from the fraction larger than 150 μm) and visual alignment of Ti/Ca values with the δ^18^O from North Greenland Ice Core Project (NGRIP). The IntCal13^41^ curve was used to calibrate radiocarbon ages and a reservoir age of 400 ± 200 years (2σ) was applied with no additional local reservoir effect (ΔR = 0). Age model was constructed using linear interpolation with the software clam 2.2^42^. Further details regarding GL-1248 age model can be found in^17^.

Quartz TL and optically stimulated luminescence (OSL) sensitivities follow the same variation trend^43^ and both variables have been used for sediment tracing analysis^44,45^. A proxy based on a single and stable mineral such as quartz is also advantageous to avoid spurious effects related to grain-size variation or post-depositional processes. Preparation and luminescence measurements of sediment samples from marine sediment core GL-1248 followed the procedure applied to marine sediment core GeoB16206-1, previously published in Ref.^21^. Sediment samples (154 in total) were collected with 2 cm wide scoops at every 10 cm from the uppermost 16 m of the marine sediment core. Samples were oven‐dried at 60 °C, precisely weighted to 0.5 g and treated with H_2_O_2_ 27% and HCl 10% to remove organic matter and calcium carbonate (CaCO_3_), respectively. Between chemical treatment, samples were washed with distilled water twice to remove the chemical reagents. We used a centrifuge to accelerate the deposition of suspended material and improve silt/clay recuperation during the distilled water washing steps. The remaining content was diluted in alcohol for luminescence measurements. Three aliquots per sample were mounted on stainless steel discs with four drops of the homogenized solution of alcohol and silt/clay sediments (close to 2 mg of sample per disc). Stokes settling time was considered to ensure that only grains of silt/clay (< 0.063 mm) were collected with the pipette used to mount the discs. GL-1248 and GeoB16206-1 luminescence measurements were performed on an automated Lexsyg Smart TL/OSL reader and a RisØ OSL/TL DA-20 reader, respectively. Both luminescence readers are equipped with blue and infrared LEDs, Hoya U-340 filters for light detection in the ultraviolet band (270–390 nm) using a photomultiplier and beta radiation sources (^90^Sr/^90^Y) with doses rate of 0.116 Gy s^−1^ (Lexsyg Smart) or 0.084 Gy s^−1^ (RisØ OSL/TL DA-20) (see in Text S1 how the results were normalized allowing comparison between the marine sediment cores measured in the different readers). Sample preparation and luminescence measurements were carried out in the Luminescence and Gamma Spectrometry Laboratory (LEGaL) of the Institute of Geosciences, University of São Paulo, Brazil. The luminescence measurement protocol applied on both marine sediment cores is described in Table 1. The used protocol recovered TL, blue OSL (BOSL) and infrared stimulated luminescence (IRSL) signals (see examples of TL, BOSL and IRSL curves on Fig. S7a–c). Since we aimed to obtain quartz TL and OSL sensitivities from a high number of fine-grained sediment samples, measurements were done in polymineral samples, without applying procedures to isolate quartz from feldspar. The IRSL at step 4 was used to reduce the contribution of feldspar grains to the OSL signal measured in step 5 (see IRSL/BOSL downcore curve in Fig. S7d). The suitability of this procedure applied to sediment provenance analysis was appraised by comparing OSL sensitivity measured in pure quartz and polymineral aliquots^44^. Additionally, the use of filters for light detection in the ultraviolet band also minimizes the effect of feldspar contamination for the OSL signal.

**Table 1:** Protocol used to measure thermoluminescence (TL) and optically stimulated luminescence (OSL) sensitivities in marine sediment cores GL-1248 and GeoB16206-1^21^.

Step	Procedure	Purpose
1	Infrared stimulation at 125 °C for 100 s	Bleach natural feldspar signals
2	Blue light stimulation at 125 °C for 100 s	Bleach natural quartz signals
3	Dose of 30 Gy (GeoB16206-1) or 15 Gy (GL-1248)	Signal regeneration
4	Infrared stimulation at 125 °C for 100 s	Bleach feldspar signals
5	Blue stimulation at 125 °C for 100 s	Measure quartz OSL for sensitivity calculation
6	Blue light stimulation at 125 °C for 100 s	Measure OSL background (0–100 s emission)
7	TL until 250 °C at 5 °C/s	Bleach natural and regenerated (step 3) TL signals
8	Dose of 30 Gy (GeoB16206-1) or 15 Gy (GL-1248)	Signal regeneration
9	TL until 250 °C at 5 °C/s	Measure TL for sensitivity calculation
10	TL until 250 °C at 5 °C/s	Measure TL background

The OSL sensitivity (Fig. S7e) was calculated from step 5 (Table 1) by integrating the first second of light emission and the last ten seconds as background (Fig. S7b). Step 6 was used to calculate the background of the total OSL emission, which was used to represent the OSL sensitivity (first second) as a percentage of the total OSL emission (0–100 s). The sensitivity representative of the 110 °C TL peak of quartz considered the 80–120 °C integration range from the TL curve (Fig. S7c) obtained through step 9 (Table 1). The 80–120 °C TL sensitivity (Fig. S7f) was calculated as a percentage of the total TL emission (0–250 °C) and using the TL curve from step 10 as background (Table 1). The mean of three measured aliquots represents the TL and OSL sensitivities of each sample.

## Supplementary Information

Supplementary Information.
