# Supplementary material for: South American precipitation dipole forced by interhemispheric temperature gradient
Source: Sci Rep. 2022 Jun 22;12:10527. doi: 10.1038/s41598-022-14495-1 (PMC9217799; doi:10.1038/s41598-022-14495-1)
Supplement: Supplementary file 1 — Supplementary Information. [file 41598_2022_14495_MOESM1_ESM.docx]

# SUPPLEMENTARY INFORMATION

# South American precipitation dipole forced by interhemispheric temperature gradient

Marília C. Campos^a^*, Cristiano M. Chiessi^b^, Valdir F. Novello^c^, Stefano Crivellari^b^, José L.P.S. Campos^a^, Ana Luiza S. Albuquerque^d^, Igor M. Venancio^d,e^, Thiago P. Santos^d^, Dayane B. Melo^f^, Francisco W. Cruz^a^, André O. Sawakuchi^a^, Vinícius R. Mendes^f^

^a^Institute of Geosciences, University of São Paulo, São Paulo, Brazil

^b^School of Arts, Sciences and Humanities, University of São Paulo, São Paulo, Brazil

^c^Department of Geosciences, University of Tübingen, Tübingen, Germany

^d^Graduate Program in Geochemistry, Fluminense Federal University, Niterói, Brazil

^e^MARUM—Center for Marine Environmental Sciences, University of Bremen, Bremen, Germany

^f^Institute of Marine Science, Federal University of São Paulo, Santos, Brazil

*Corresponding author: marilia.carvalho.campos@usp.br

# Supplementary texts

**Text S1.** Marine sediment cores GL-1248 and GeoB16206-1 were analyzed in different luminescence readers, i.e. automated Lexsyg Smart TL/OSL reader and RisØ OSL/TL DA-20 reader, respectively, and using different regeneration doses (see the methods description for details regarding measurements). In order to avoid machine artifacts and the influence of dose size on sensitivity, we normalized the thermoluminescence (TL) sensitivity from both marine sediment cores (TL sensitivity normalized = (x-x_mean_)/σ_x_, where x is the TL sensitivity value, x_mean_ refers to the mean and σ to the standard deviation of all TL sensitivity values). For practical reasons, we choose to refer to the TL sensitivity normalized data from GL-1248 and GeoB16206-1 composite record as simply “TL sensitivity”.

In order to obtain the composite timeseries (i.e. GL-1248 and GeoB16206-1 merged time series), we: (i) interpolated both timeseries into a uniform resolution (i.e. 500 yr); (ii) standardized the resultant timeseries considering only the mean and standard deviation of the overlapping period (i.e. subtracting the mean and dividing by the standard deviation of the overlapping period); and (iii) calculated the averaged timeseries and inverted the standardization (by multiplying the resultant timeseries by the standard deviation and adding the mean of the longer timeseries). Since the uppermost part of GL-1248 timeseries (i.e. ca. 15-0 ka BP) has coarser resolution than the equivalent period in GeoB16206-1 timeseries, only the overlapping period between 31 and 27 ka BP was considered to perform the composite record (Fig. S1). We followed the Monte-Carlo age-depth model and composite timeseries procedures described in ref. ^1^.

The chronological timeseries of the GL-1248 and GeoB16206-1 composite record was sampled in a higher resolution (i.e. 500 yrs) than the TL sensitivity timeseries. Thus, to obtain the corresponding age of a given TL sensitivity datapoint, a linear regression was performed over pairs of chronological age-depth datapoints. To take into account the age uncertainty of the chronological series, 1,000 Monte-Carlo simulations were performed over the records using a pseudo-random gaussian number generator to simulate the age uncertainty, resulting in 1,000 possible timeseries for each record (Fig. S1a).

**Text S2.** The use of quartz TL sensitivity from marine sediment cores as a proxy for past precipitation changes over the adjacent continent was first described in ref ^2^. By analyzing samples from marine sediment core GeoB16206-1 and continental sediments derived from the Parnaíba River drainage basin, the authors could accurately constrain precipitation changes over northern northeastern Brazil (NEB) for the last 30 ka. They showed that the sensitivity of the 110^o^C TL peak, and also the optically stimulated luminescence (OSL) sensitivity from grains of quartz deposited off the Parnaíba River drainage basin are modulated by changes in the contribution of different sediment sources within the basin. The Parnaíba River drainage basin is mainly composed of two domains: (i) terrains of Precambrian igneous and metamorphic rocks in the upstream headings, under a Caatinga biome with semiarid climate and (ii) Paleozoic-Mesozoic sedimentary rocks in the middle and downstream sectors of the drainage basin, under a Cerrado biome with tropical climate (Fig. S2). Sediments produced in these two domains have specific TL and OSL signatures, where soils from the headings provide quartz grains with higher TL and OSL sensitivities (region I in Fig. S2). Thus, increased (decreased) precipitation over the headings is tracked by enhanced input of quartz grains with higher TL and OSL sensitivities off the Parnaíba River drainage basin, i.e. increasing (decreasing) the TL and OSL sensitivities of marine sediments. Once the Parnaíba River drainage basin has a latitudinal distribution with headings located under the descending branch of the regional Walker circulation and the southernmost seasonal reach of the ITCZ, it is possible to capture changes in Walker circulation and ITCZ-related precipitation. The TL and OSL sensitivities of quartz grains depend only on the sediments transported through the Parnaíba River drainage basin. Thus, they can trace the water discharge independently of marine processes such as sea-level changes and biogenic carbonate production.

Despite similar general trends (Fig. S7e and f), the TL and OSL sensitivities showed some differences in the studied marine sediment core. This was attributed to the higher influence of remaining feldspar signals to the OSL emission. The 110^o^C TL peak would be less susceptible to feldspar contamination because feldspar TL in the ultraviolet band increases only after 150^o^C^3^. Thus, we choose to focus our interpretation on TL sensitivity.

**Text S3.** The loss of strong insolation control between ca. 55 and 20 ka BP suggests that other factors may have contributed to control precipitation over tropical South America (SA)^4^. This period occurred under full glacial boundary conditions that may increase the magnitude of the eccentricity forcing relative to precessional forcing over tropical hydroclimate (Fig. S5)^4-7^. Under full glacial conditions (low eccentricity), ice volume buildup in the Northern Hemisphere (NH) resulting in warmth of the Southern Hemisphere (SH) relative to the NH. It shifted the maximum sea-surface temperatures to the south of Equator, increasing ocean-continent moisture transport and strengthening the Walker circulation. In turn, convection over the SAMS core region became stronger, increasing precipitation over W Amazonia (Fig. S5b) as well as over eastern South America (Fig. S5a) and decreasing precipitation over NEB (Fig. S5c).

**Text S4.** On short-term, millennial-scale changes in hydroclimate (e.g. physical erosion, chemical weathering and river transport) over the northeastern Brazil were the main driver of terrigenous input to the adjacent continental margin imprinting positive anomalies in the Ti/Ca, Fe/Ca, Fe/K, and Al/Si ratios off NEB^8-13^. At this timescale, changes in relative sea-level proved not to play an important role in controlling elemental ratios variability^14,15^. Instead, on glacial-interglacial timescale, sea-level changes seem to be the most important driver of terrigenous input to off NEB. The imprint of sea-level signal over the elemental ratios is demonstrated by their clear inverse relationship (Figs. 5 and S6a-c). For Ti/Ca and Fe/Ca, the controlling forcings are clearly stated, considering the predominant terrigenous (marine) origin of Ti and Fe (Ca). However, since K, Al, and Si are predominantly terrigenous sourced, the inverse relationship between sea-level and Fe/K and Al/Si ratios is less obvious.

Our 113 ka-long TL sensitivity record shows that precession drove NEB precipitation, where increased (decreased) precipitation took place during minimum (maximum) austral summer insolation. Curiously, while the TL sensitivity clearly recorded precession driven changes in precipitation, the elemental ratios only respond to shorter (e.g. millennial-scale events) and/or longer (e.g. glacial-interglacial cycles) changes indicating that the variety of factors that can influence major elements records (e.g. relative sea-level, chemical weathering, source area) may overprint the precession signal. Importantly, while the TL sensitivity is obtained from quartz, which is an abundant, resistant and relatively lighter mineral, the elemental ratios represent the bulk sediment and depend on several mineral phases. Thus, the great advantage of looking at a specific mineral and grain size fraction is the possibility of isolating variables. For instance, the TL sensitivity variability in marine sediment cores GL-1248 and GeoB16206-1 is clearly related to precipitation changes over the source area within the Parnaíba River drainage basin^2^. The sediment provenance changes interpreted through the TL sensitivity signal are supported by the εNd(0) record from GL-1248^13^ (Fig. S6e and f). Despite the very low temporal resolution of the εNd(0) record, both suggest increased precipitation over the headings during minimum austral summer insolation. These changes in sediment source over-time may explain why elemental ratios Fe/K and Al/Si do not record the precessional precipitation signal. For instance, the use of Fe/K as a proxy for chemical weathering assumes the same parental rock going under different climates, which was not the case. At the same time, the Al/Si, which is generally associated with grain size changes^16^, presents a direct correlation to Fe/K and, in turn, both ratios are inversed correlated to relative sea-level. Sea-level controls the distance between the river mouth and the marine sediment core site, i.e. longer (shorter) distance increases (decreases) the oceanic transport route promoting grain size and mineral (related to different densities) sorting. Thus, the combined effect of sediment source and sea-level changes seems to obliterate the precessional signal in elemental ratios related to NEB.

In addition, upper water column stratification data (based on the δ^18^O of two planktonic foraminifera species) from marine sediment core GL-1248^17^ agree with increased precipitation during periods of minimum austral summer insolation (Fig. S6d). Despite the authors had originally attributed the stratification changes to processes related to the boreal summer insolation and southeastern trade winds, the stronger upper stratification (which occurred coevally with minimum austral summer insolation) could also be favored by increased precipitation as demonstrated by physical oceanography (e.g. ^18^) and paleoceanography (e.g. ^19^) studies.

Finally, a recently published GL-1248 palynological record^20^ did not either respond to the precession signal. However, we do not expect it would respond to precession since this record is highly influenced by the lowland vegetation that, in turn, may not be sensitive to precipitation changes over the headings of the basin. Indeed, the authors suggested that the signal is mainly controlled by the adjacent Atlantic sea-surface temperature throughout its influence on moisture availability.

# Supplementary figures


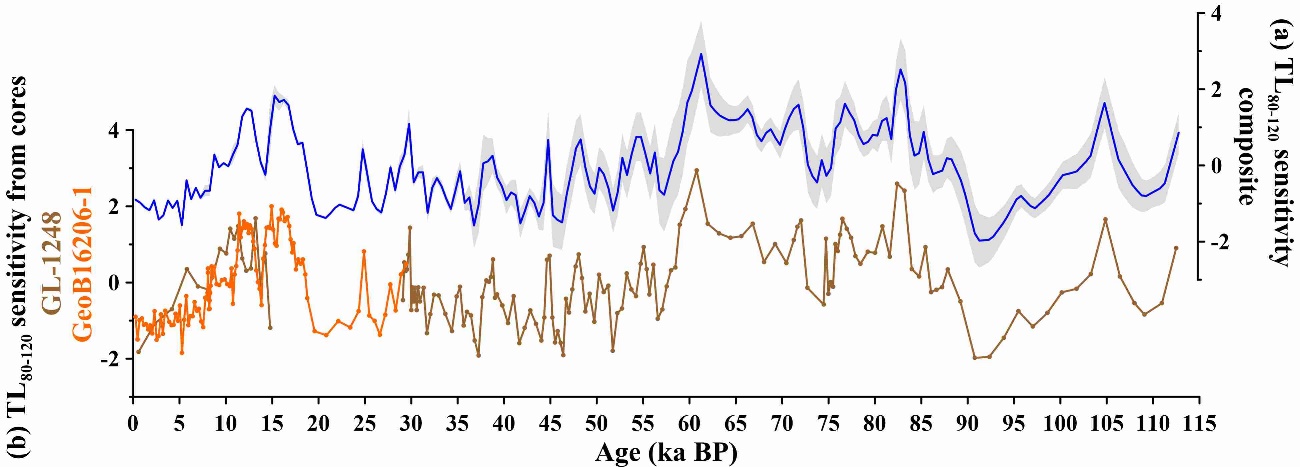


**Figure S1.** **(a)** GeoB16206-1/GL-1248 composite record of thermoluminescence (TL_80–120_) sensitivity of the 110^o^C peak. **(b)** GeoB16206-1^2^ (orange) and GL-1248 (gold) records. The shaded envelop in panel (a) represents the standard deviation of 1,000 Monte-Carlo simulations performed with an age-depth linear model (see supplementary Text S1).


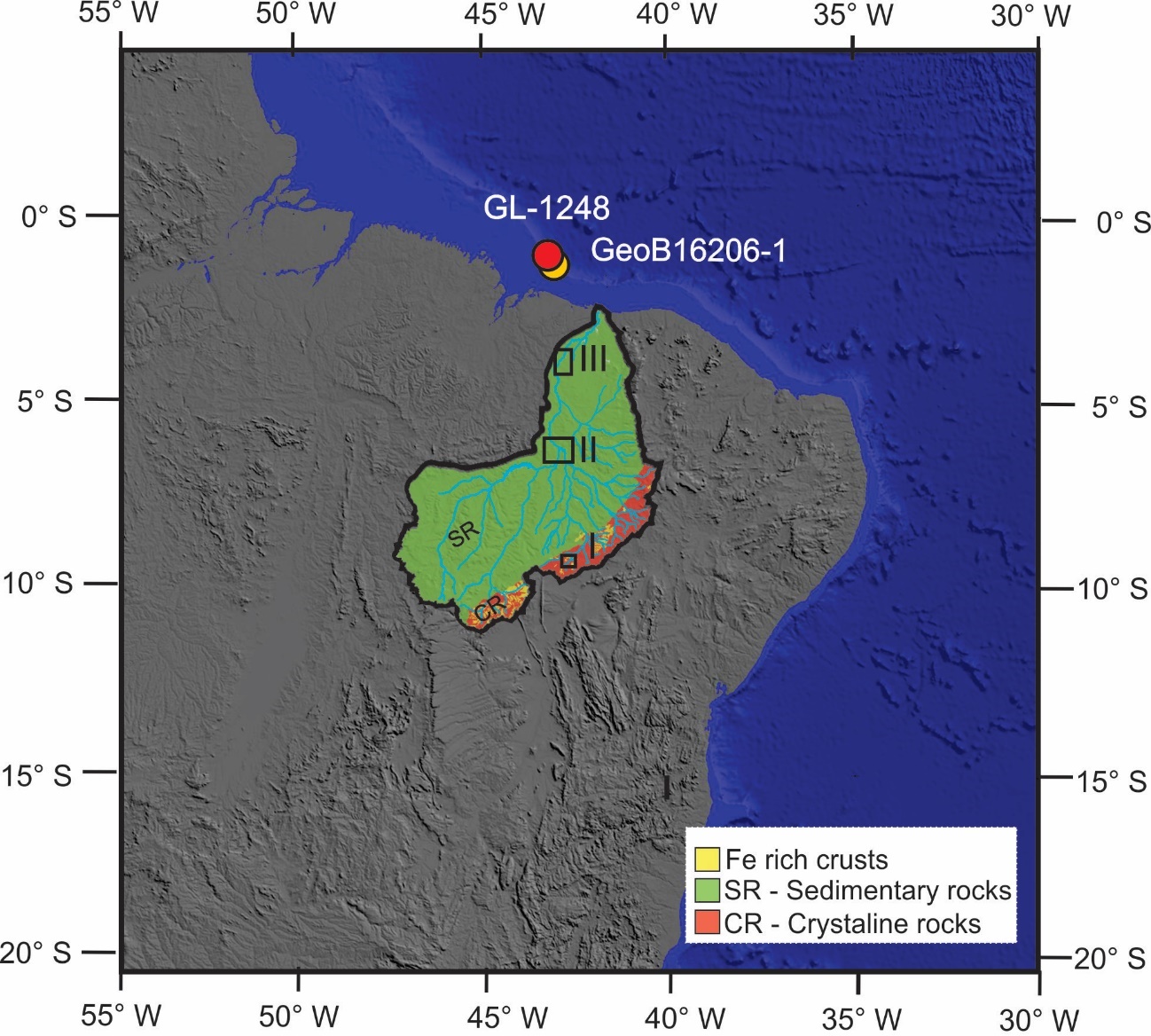


**Figure S2.** Simplified geological map of the Parnaíba River drainage basin (main tributaries depicted by the blue lines), location of the drainage basin sample sites (black rectangles: I - Porcos Lake; II and III - medium and low Parnaíba River, respectively) and location of marine sediment cores GL-1248 and GeoB16206-1 (adapted from ref. ^2^). Parnaíba River drainage basin main domains are terrains of Precambrian igneous and metamorphic rocks in the upstream headings and Paleozoic-Mesozoic sedimentary rocks in the middle and downstream sectors of the drainage basin.


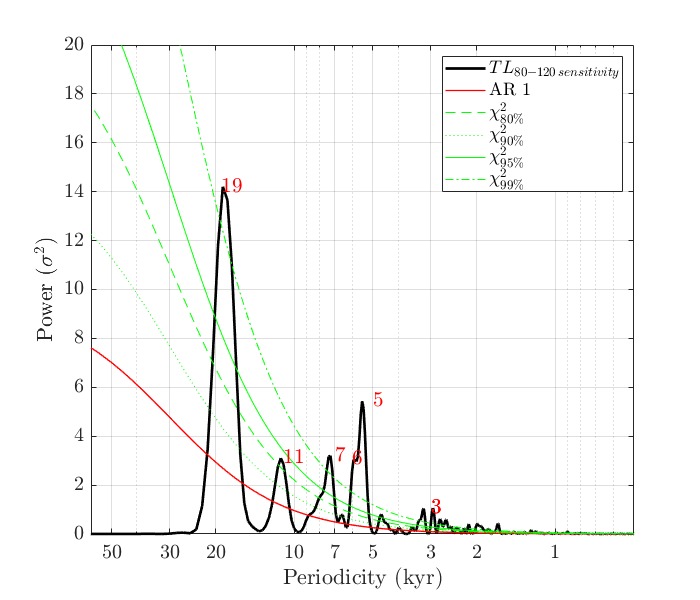


**Figure S3.** Power density estimate of the thermoluminescence (TL_80–120_) sensitivity of the 110^o^C peak from marine sediment cores GL-1248 and GeoB16206-1^2^ with the REDFIT algorithm^21^. Periods are represented in thousand years (kyr) and red numbers are the peak values. Red line (AR 1) refers to the red-noise spectrum. The green lines refer to the false-alarm levels at 99% (x^2^_99%_), 95% (x^2^_95%_), 90% (x^2^_90%_), and 80% (x^2^_80%_). Bandwidth is 1.412 10^-2^ kyr^-1^.


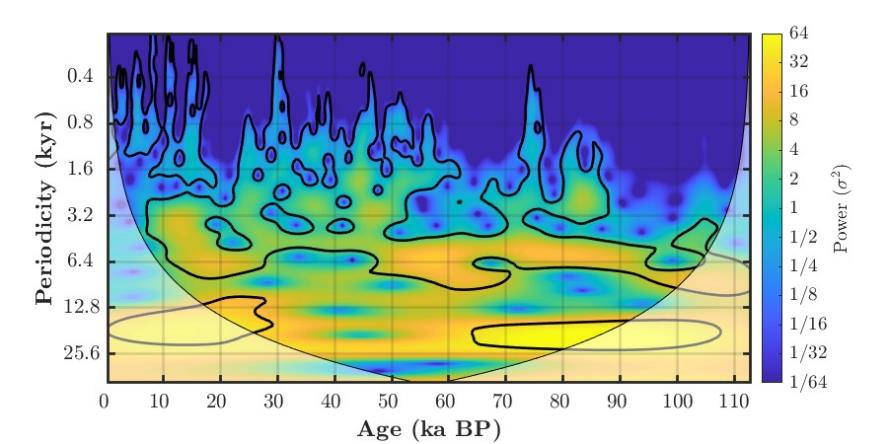


**Figure S4.** Morlet wavelet scalogram of the thermoluminescence (TL_80–120_) sensitivity of the 110^o^C peak from marine sediment cores GL-1248 and GeoB16206-1^2^. The thick black contour designates the 5% significance level against red noise hypothesis and the Cone of Influence (COI) where edge effects might distort the analysis is shown as a lighter shade.


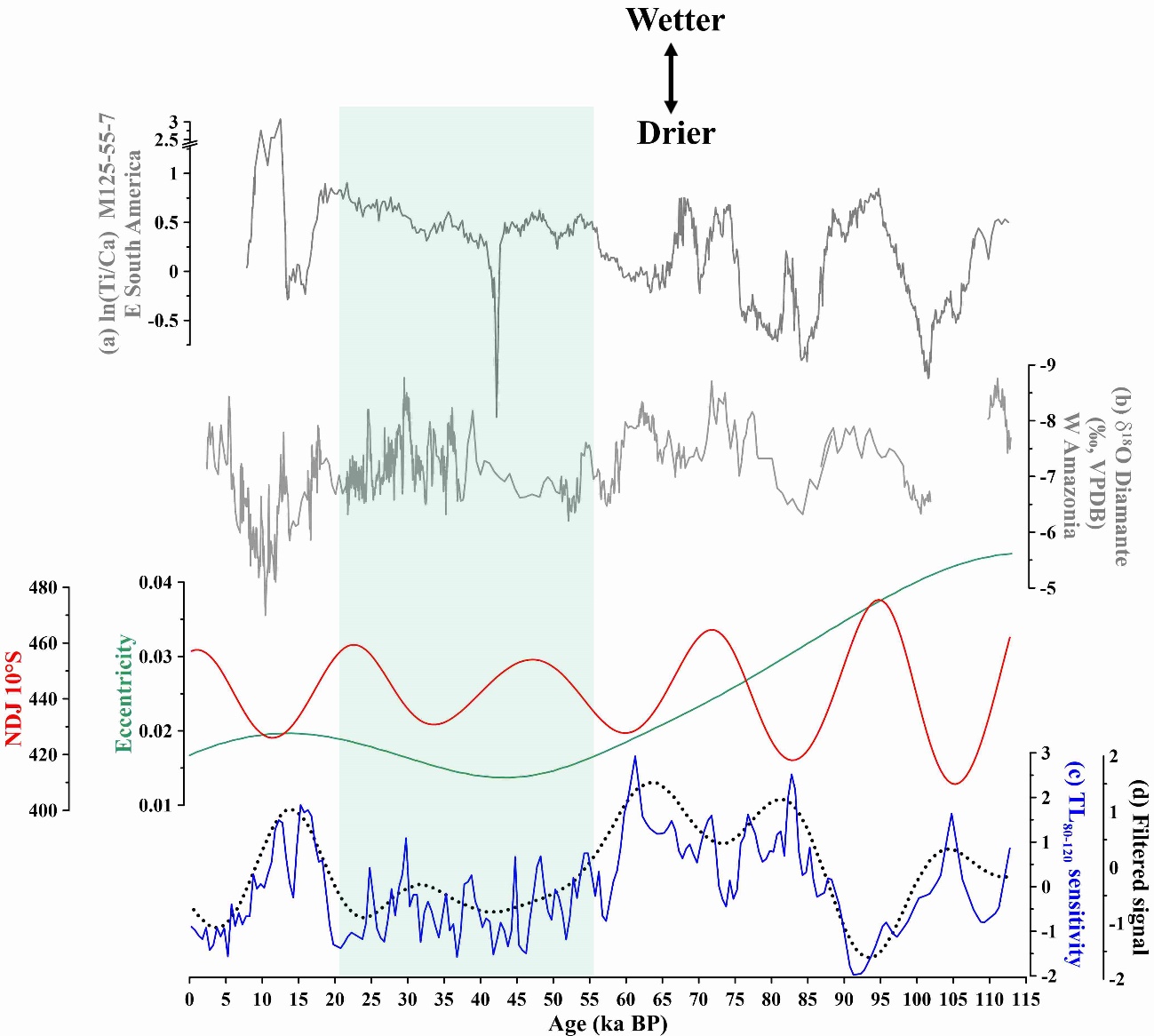


**Figure S5.** Precipitation records during the period 55-20 ka BP (green rectangle). **(a)** ln(Ti/Ca) from marine sediment core M125-55-7, off E South America^22^ (20.4°S, 38.6°W; the axis was broken from 1.25 to 2.5). **(b)** Speleothem stable oxygen isotopic (δ^18^O) records from Diamante Cave, western (W) Amazonia (ca. 5°S, 77°W)^23^. **(c)** Thermoluminescence (TL_80–120_) sensitivity of the 110^o^C peak from marine sediment cores GL-1248 (0.92°S, 43.40°W) and GeoB16206-1 (1.58°S, 43.02°W)^2^, off northern NE Brazil (NEB, this study). **(d)** TL_80–120_ sensitivity record filtered in 0.026 ka frequency (19 ka, FIR filter) within software PAST v4.03^24^. Green and red curves are eccentricity and November–January (NDJ) insolation (W/m^2^) at 10°S^25^, respectively.


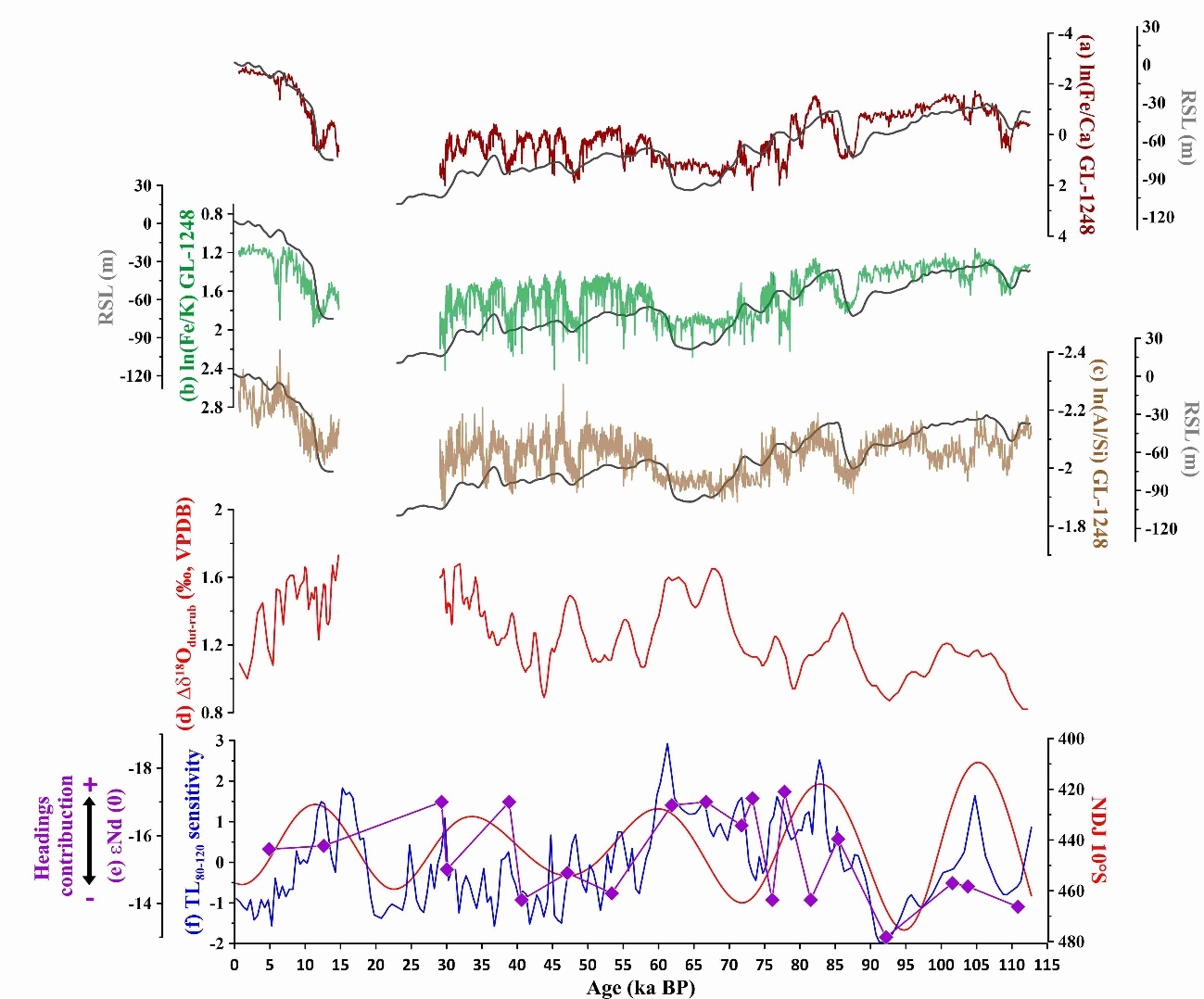


**Figure S6.** GL-1248 records compared to relative sea-level (RSL) probability maximum (dark grey)^14^ and November–January (NDJ) insolation (W/m^2^) at 10°S^25^. **(a)** GL-1248 ln(Fe/Ca)^13^; **(b)** GL-1248 ln(Fe/K)^13^. **(c)** GL-1248 ln(Al/Si)^13^. **(d)** GL-1248 stable oxygen isotopic (δ^18^O) record based on two species of planktonic foraminifera (i.e. δ^18^O *Neogloboquadrina dutertrei* minus δ^18^O *Globigerinoides ruber white, i.e.* Δδ^18^Odut-rub)^17^. **(e)** GL-1248 εNd data^13^. **(f)** GL-1248 (this study) and GeoB16206-1^2^ thermoluminescence (TL_80–120_) sensitivity of the 110^o^C peak.

**
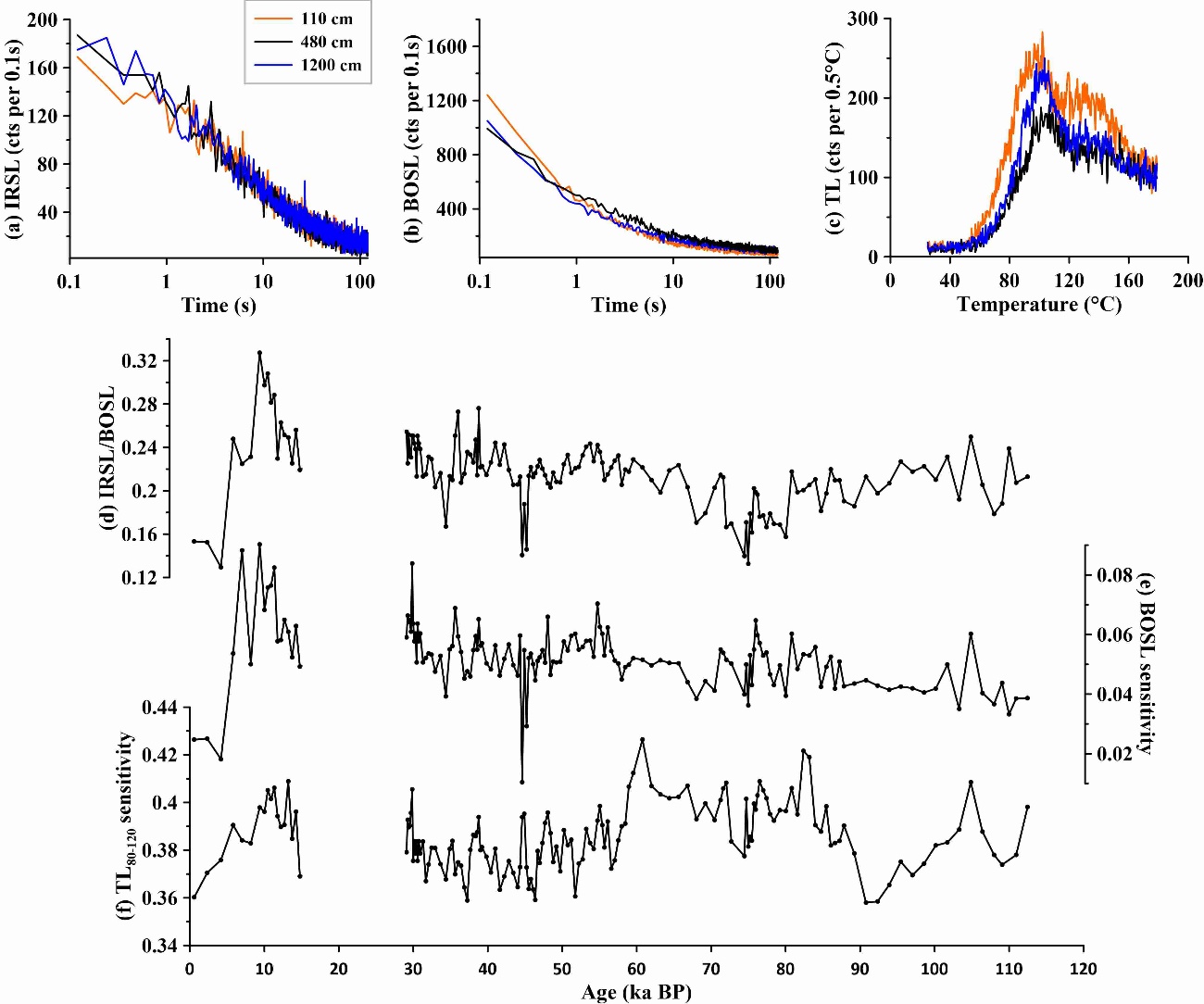
Figure S7.** Panels (a) to (c) show examples of marine sediment core GL-1248 **(a)** infrared stimulated luminescence (IRSL) decay curves (x axis in logarithmic scale), **(b)** blue optically stimulated luminescence (BOSL) decay curves (x axis in logarithmic scale) and **(c)** thermoluminescence (TL) glow curves measured in steps 4, 5, and 9, respectively, of the protocol described in Table 1. Orange, black and blue curves represent one aliquot measured from GL-1248 depths 110, 480 and 1200 cm, respectively. Panels (d) to (f) show marine sediment core GL-1248 downcore data for **(d)** IRSL/BOSL, **(e)** BOSL sensitivity and **(f)** TL_80-120_ sensitivity of peak 110°C.

**References**

1 Campos, J. *et al.* Coherent South American Monsoon variability during the last millennium revealed through high‐resolution proxy records. *Geophysical Research Letters* **46**, 8261-8270 (2019).

2 Mendes, V. R. *et al.* Thermoluminescence and optically stimulated luminescence measured in marine sediments indicate precipitation changes over northeastern Brazil. *Paleoceanography and Paleoclimatology* **34**, 1476-1486 (2019).

3 Blair, M. W., Yukihara, E. G. & McKeever, S. W. S. Experiences with single-aliquot OSL procedures using coarse-grain feldspars. *Radiation Measurements* **39**, 361-374, doi:<https://doi.org/10.1016/j.radmeas.2004.05.008> (2005).

4 Cruz, F. W. *et al.* Evidence of rainfall variations in Southern Brazil from trace element ratios (Mg/Ca and Sr/Ca) in a Late Pleistocene stalagmite. *Geochimica et Cosmochimica Acta* **71**, 2250-2263 (2007).

5 Clement, A. C., Hall, A. & Broccoli, A. The importance of precessional signals in the tropical climate. *Climate Dynamics* **22**, 327-341 (2004).

6 Cruz, F. W. *et al.* Insolation-driven changes in atmospheric circulation over the past 116,000 years in subtropical Brazil. *Nature* **434**, 63-66 (2005).

7 Mosblech, N. A. *et al.* North Atlantic forcing of Amazonian precipitation during the last ice age. *Nature Geoscience* **5**, 817-820 (2012).

8 Arz, H. W., Pätzold, J. & Wefer, G. Correlated millennial-scale changes in surface hydrography and terrigenous sediment yield inferred from last-glacial marine deposits off northeastern Brazil. *Quaternary Research* **50**, 157-166 (1998).

9 Campos, M. C. *et al.* A new mechanism for millennial scale positive precipitation anomalies over tropical South America. *Quaternary Science Reviews* **225**, 105990 (2019).

10 Jaeschke, A., Rühlemann, C., Arz, H., Heil, G. & Lohmann, G. Coupling of millennial‐scale changes in sea surface temperature and precipitation off northeastern Brazil with high‐latitude climate shifts during the last glacial period. *Paleoceanography* **22** (2007).

11 Mulitza, S. *et al.* Synchronous and proportional deglacial changes in Atlantic meridional overturning and northeast Brazilian precipitation. *Paleoceanography* **32**, 622-633 (2017).

12 Zhang, Y. *et al.* Origin of increased terrigenous supply to the NE South American continental margin during Heinrich Stadial 1 and the Younger Dryas. *Earth and Planetary Science Letters* **432**, 493-500 (2015).

13 Sousa, T. A. *et al.* Changes in sedimentary provenance and climate off the coast of Northeast Brazil since the Last Interglacial. *Marine Geology*, 106454 (2021).

14 Grant, K. *et al.* Rapid coupling between ice volume and polar temperature over the past 150,000 years. *Nature* **491**, 744-747 (2012).

15 Siddall, M., Rohling, E. J., Thompson, W. G. & Waelbroeck, C. Marine isotope stage 3 sea level fluctuations: Data synthesis and new outlook. *Reviews of Geophysics* **46** (2008).

16 Govin, A. *et al.* Distribution of major elements in Atlantic surface sediments (36 N–49 S): Imprint of terrigenous input and continental weathering. *Geochemistry, Geophysics, Geosystems* **13** (2012).

17 Venancio, I. *et al.* Millennial‐to Orbital‐Scale Responses of Western Equatorial Atlantic Thermocline Depth to Changes in the Trade Wind System Since the Last Interglacial. *Paleoceanography and Paleoclimatology* **33**, 1490-1507 (2018).

18 Mignot, J., de Boyer Montégut, C., Lazar, A. & Cravatte, S. Control of salinity on the mixed layer depth in the world ocean: 2. Tropical areas. *Journal of Geophysical Research: Oceans* **112** (2007).

19 Portilho-Ramos, R. *et al.* Coupling of equatorial Atlantic surface stratification to glacial shifts in the tropical rainbelt. *Scientific reports* **7**, 1561 (2017).

20 Piacsek, P. *et al.* Reconstruction of vegetation and low latitude ocean-atmosphere dynamics of the past 130 kyr, based on South American montane pollen types. *Global and Planetary Change* **201**, 103477 (2021).

21 Schulz, M. & Mudelsee, M. REDFIT: estimating red-noise spectra directly from unevenly spaced paleoclimatic time series. *Computers & Geosciences* **28**, 421-426 (2002).

22 Hou, A. *et al.* Insolation and Greenhouse Gas Forcing of the South American Monsoon System Across Three Glacial‐Interglacial Cycles. *Geophysical Research Letters* **47**, e2020GL087948 (2020).

23 Cheng, H. *et al.* Climate change patterns in Amazonia and biodiversity. *Nature communications* **4**, 1-6 (2013).

24 Hammer, Ø., Harper, D. A. & Ryan, P. D. PAST: Paleontological statistics software package for education and data analysis. *Palaeontologia electronica* **4**, 9 (2001).

25 Laskar, J., Fienga, A., Gastineau, M. & Manche, H. La2010: a new orbital solution for the long-term motion of the Earth. *Astronomy & Astrophysics* **532**, A89 (2011).
